# Chemical Labeling Assisted Detection and Identification of Short Chain Fatty Acid Esters of Hydroxy Fatty Acid in Rat Colon and Cecum Contents

**DOI:** 10.3390/metabo10100398

**Published:** 2020-10-08

**Authors:** Siddabasave Gowda B. Gowda, Divyavani Gowda, Chongsheng Liang, Yonghan Li, Kentaro Kawakami, Satoru Fukiya, Atsushi Yokota, Hitoshi Chiba, Shu-Ping Hui

**Affiliations:** 1 Faculty of Health Sciences, Hokkaido University, Kita-12 Nishi-5, Kita-Ku, Sapporo 060-0812, Japan; siddabasavegowda.bommegowda@hs.hokudai.ac.jp (S.G.B.G.); divyavani@hs.hokudai.ac.jp (D.G.); 2 Graduate School of Global Food Resources, Hokkaido University, Kita-9, Nishi-9, Kita-Ku, Sapporo 060-0809, Japan; 3 Graduate School of Health Sciences, Hokkaido University, Kita-12 Nishi-5, Kita-Ku, Sapporo 060-0812, Japan; liangchongsheng@gmail.com (C.L.); liyonghan846@gmail.com (Y.L.); 4 Research Faculty of Agriculture, Hokkaido University, Kita-9 Nishi-9, Kita-ku, Sapporo 060-8589, Japan; k-kawakami@chem.agr.hokudai.ac.jp (K.K.); s-fukiya@chem.agr.hokudai.ac.jp (S.F.); yokota@chem.agr.hokudai.ac.jp (A.Y.); 5 Department of Nutrition, Sapporo University of Health Sciences, Nakanuma Nishi-4-3-1-15, Higashi-Ku, Sapporo 007-0894, Japan; chiba-h@sapporo-hokeniryou-u.ac.jp

**Keywords:** FAHFAs, Chemical labeling, DMED, High-fat diet, murine model, LC-MS

## Abstract

Branched fatty acid esters of hydroxy fatty acids (FAHFAs) are novel endogenous lipids with important physiological functions in mammals. We previously identified a new type of FAHFAs, named short-chain fatty acid esterified hydroxy fatty acids (SFAHFAs), with acetyl or propyl esters of hydroxy fatty acids of carbon chains, C ≥ 20. However, sensitive determination of SFAHFAs is still a challenge, due to their high structural similarity and low abundance in biological samples. This study employs one-step chemical derivatization following total lipid extraction using 2-dimethylaminoethylamine (DMED) for enhanced detection of SFAHFAs. The labeled extracts were subjected to ultrahigh performance liquid chromatography coupled to linear ion trap quadrupole-Orbitrap mass spectrometry (UHPLC/LTQ-Orbitrap MS). Our results demonstrated that the detection sensitivities of SFAHFAs increased after DMED labeling, and is highly helpful in discovering six additional novel SFAHFAs in the cecum and colon contents of WKAH/HKmSlc rats fed with normal and high-fat diet (HFD). The identified DMED labeled SFAHFAs were characterized by their detailed MS/MS analysis, and their plausible fragmentation patterns were proposed. The concentrations of SFAHFAs were significantly reduced in the cecum of HFD group compared to the control. Hence, the proposed method could be a promising tool to apply for the enhanced detection of SFAHFAs in various biological matrices, which in turn facilitate the understanding of their sources, and physiological functions of these novel lipids.

## 1. Introduction

Lipids play important roles in biology, including structural components of cellular membranes, acting as energy storage and signaling molecules that contribute to the regulation of many cellular processes. Fatty acids and their derivatives serve as bioactive mediators in obesity and associated disorders [1,2,3]. Fatty acid esters of hydroxyl fatty acids (FAHFAs) are the new class lipids though they first identified in the European cabbage butterfly (named as mayolenes) [4], but recently received considerable attention after analyzing them in mammalian white adipose tissue with potential anti-inflammatory and anti-diabetic activities [5,6,7]. FAHFAs are potentially anti-diabetic and anti-inflammatory molecules, as they enhance the insulin secretion by up taking blood glucose levels and reduce the inflammation associated with obesity [5]. Dietary intake of polyunsaturated fatty acid-containing FAHFAs known to stimulate immune cells and inhibit the inflammation by limiting the macrophage activation [6]. Oral administration of FAHFAs, such as branched palmitic acid esters of hydroxy stearic acid, attenuated the severity of inflammatory bowel disease by decreasing the growth of pro-inflammatory T lymphocytes [8]. More recently, we have shown that Eicosapentaenoic acid-derived FAHFAs as Nrf2 activators and promising antioxidants [9]. 

Despite recognizing these important functions in the biological process, the physiological function and molecular regulation mechanism of FAHFAs are still unclear. Given the large structural diversity, FAHFAs, functional studies depend on the sensitive analytical method for their determination. A variety of regio-isomers have been identified with an esterified hydroxyl group position at 5, 7, 8, 9, 10, 11, 12, and 13 [5,10], but there are limited reports on C2 or C3 [11] although C2 and C3 hydroxy fatty acids are widely spread in nature. We have recently identified a new type of lipids to the FAHFAs family named short-chain fatty acid esters of hydroxyl fatty acids (SFAHFAs) with ester linkage at C2 in the various tissues, plasma, and contents of the gastrointestinal tract [12]. Several liquid-chromatography mass spectrometry (LC-MS) based analytical methods were established to determine FAHFAs in foods and various biological matrices [5,6,10,13,14]. However, FAHFAs are low abundant and these techniques are limited by the low detection sensitivity and poor isomer separation. An effort to overcome these problems by chemical labeling experiments using n-[4-(aminomethyl) phenylpyridinium] (AMPP) [15], 2-dimethylaminoethylamine (DMED) [10,16] were established. In particular, DMED labeling of carboxyl moiety of FAHFAs was found to be advantageous because of enhanced detection sensitivities from 7–72 folds [10]. Moreover, the DMED labeling technique improved the identification of many isomeric species, and their in-silico generated libraries were established [16].

In our previous study, we have uncovered about ten new SFAHFAs, excluding some having unseparated structural isomers by untargeted LC-MS analysis with MS^n^ measurements [12]. These molecular species are low in abundance, but were significantly altered with the high-fat diet in the murine model. In this study, to improve SFAHFAs detection sensitivities and to discover more unknown molecular species, we have applied the DMED-labeling technique to the total lipid extracts of rat intestinal contents. The applied DMED labeling technique allows the determination of additional six new SFAHFAs with high mass accuracy by using ultra-high-performance liquid chromatography coupled linear ion trap quadrupole-Orbitrap mass spectrometry (UHPLC/LTQ Orbitrap MS) in positive ion electrospray ionization mode with collision-induced dissociation (CID). The detection sensitivities of this technique improved with approximately 3–4 folds higher compared to our previous method. The relative concentrations of DMED-labeled-SFAHFAs were measured in colon and cecum contents of rats fed under normal diet and high-fat diet.

## 2. Results and Discussion

### 2.1. Chemical Labeling Assisted Detection and Identification of SFAHFAs

**Figure 1:**
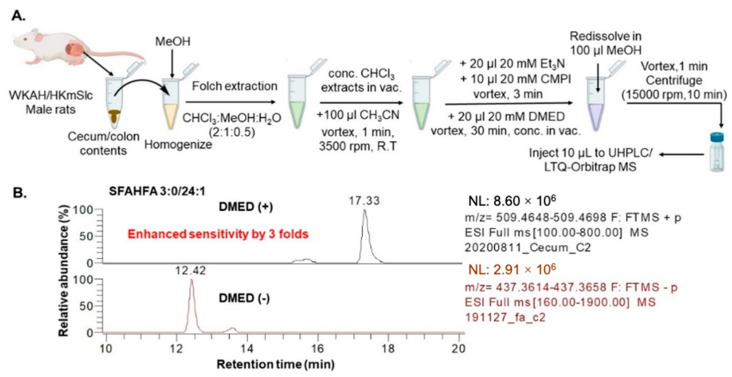
(**A**) Extraction and 2-dimethylaminoethylamine (DMED)-labeling workflow for detection of short-chain fatty acid esterified hydroxy fatty acids (SFAHFAs) (**B**) Extracted ion chromatograms (EICs) of a representative SFAHFA 3:0/24:1 molecular species with (in positive mode) and without DMED labeling (in negative mode).

In this study, we applied a chemical labeling-assisted untargeted LC-MS method for enhanced detection of SFAHFAs, which were identified earlier by our group. To this end, Folch extraction (coupled with DMED labeling) was employed (Figure 1A). The DMED derivatization technique was established and previously applied for the sensitive determination of long-chain FAHFAs by Feng Y.Q. et al. [10,11]. However, this the first report to apply the technique to a homologous series of short-chain hydroxy fatty acid esters with minor modifications. Although high efficiency was found at 40 °C with a duration of 1hr derivatization for FAHFAs DMED-labeling [10], we employed mild conditions, such as reduce the temperature to room conditions and shorten the derivatization time to 30 min, to avoid hydrolysis of short-chain fatty acids from the SFAHFAs. 

**Figure 2:**
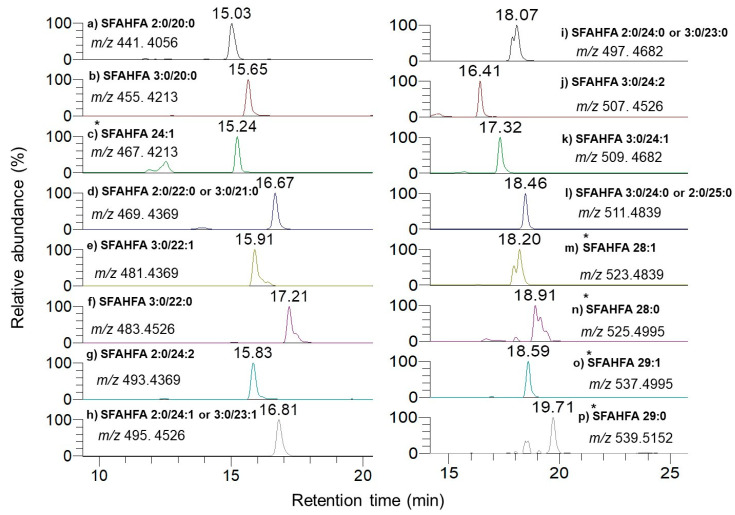
Extracted ion chromatograms of DMED-labeled SFAHFAs (* represents the SFAHFAs for which MS/MS data was not acquired).

The extracted ion chromatograms (EICs) of the previously identified SFAHFA 3:0/24:1 without DMED labeling (intensity: 2.9 × 10^6^) and same sample with the DMED labeling (intensity: 8.6 × 10^6^) were shown in Figure 1B. As the data shows, signal intensity of DMED-SFAHFA is increased by almost three folds higher compared to unlabeled SFAHFA. Though this change is small, it contributes largely to the detection of other minor SFAHFAs, which were unidentified in our previous study [12]. Table 1 shows the list of SFAHFAs identified after DMED labeling, which includes additional six new molecular species which were identified in this study. The EICs of all the SFAHFAs with their *m*/*z* and retention time values are shown in Figure 2. The SFAHFAs were putatively identified according their retention time behavior on a C18 column and on their accurate mass values. Generally, shorter saturated carbon chain SFAHFAs elutes first followed by the longer chain with a slightly earlier elution of their mono or di unsaturated ones. The long-chain hydroxy fatty acids (C ≥ 20), including odd ones esterified with short-chain fatty acids identified in this study, are known to be biosynthesized in the gut and incorporated into complex lipids [17].

### 2.2. Annotation of DMED-labeled-SFAHFAs by MS/MS Analysis

**Figure 3:**
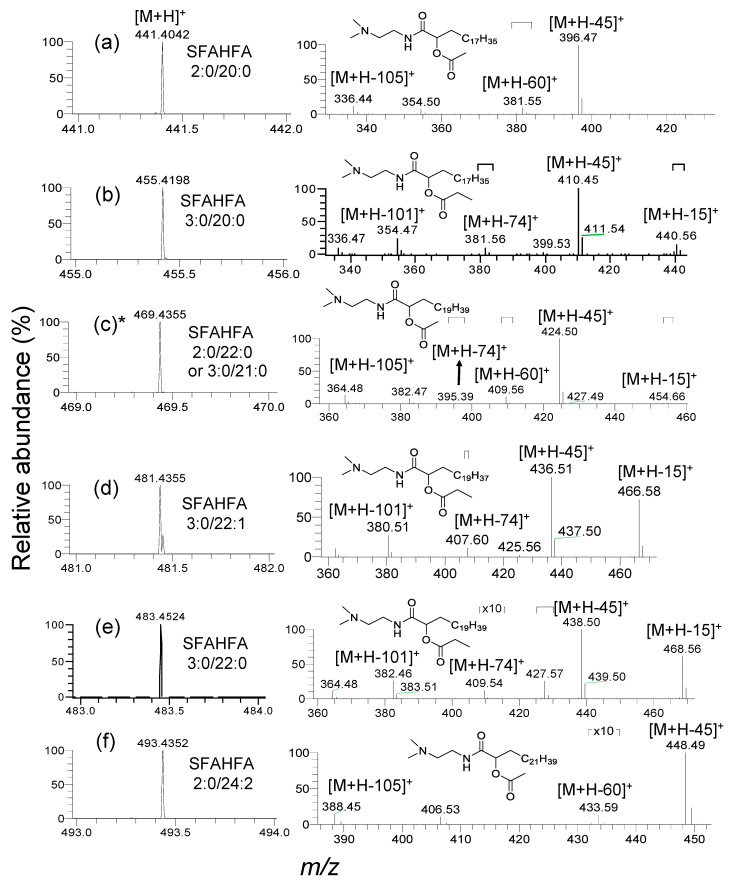
Mass spectra (MS^1^) of DMED-labeled SFAHFAs and their acquired MS/MS spectra along with tentative chemical structures (**a**–**f**). (* represents the isomeric mixtures).

**Figure 4:**
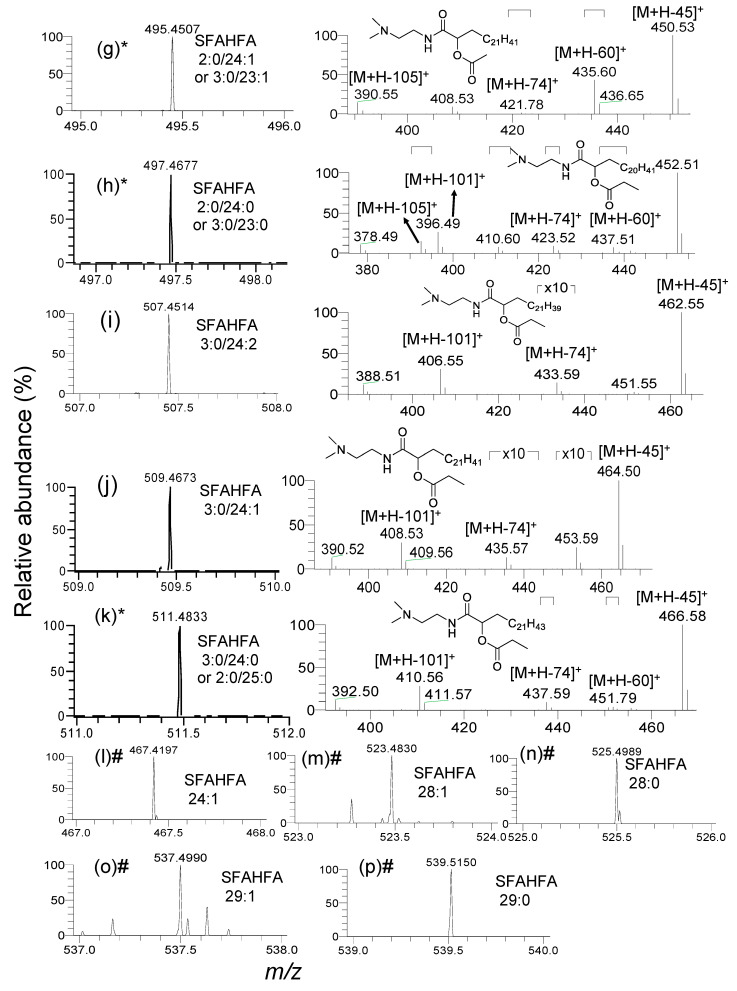
Mass spectra (MS^1^) of DMED-labeled SFAHFAs and their acquired MS/MS spectra along with tentative chemical structures (**g**–**p**). (* represents the isomeric mixtures and # represents the SFAHFAs for which MS/MS are not shown).

The injection of DMED-labeled-lipid extracts of rat cecum and colon contents to the LC-MS provides the heuristic information on MS/MS fragmentation patterns of DMED-labeled-SFAHFAs. The MS^1^ and CID MS/MS spectra of the DMED labeled SFAHFAs identified in the study were shown in Figure 3 and Figure 4. Mainly, there are two types of SFAHFAs with acetic acid or propionic acid hydroxy fatty acid esters were found to be abundant. For example, the molecular ion [M+H]^+^ of DMED-labeled-SFAHFA 2:0/20:0 (Figure 3a) as experimental *m*/*z* 441.4035 (Theoretical *m*/*z*: 441.4056, mass error: −3.1 ppm) will lose a neutral molecule of dimethylamine (*m*/*z* 45 Da) to produce a predominant ion of *m*/*z* 396. The neutral loss of the *m*/*z* 60 Da from the molecular ion suggests the acylated side chain of long-chain fatty acid is acetic acid. So far, the FAHFAs identified are esterified at carbon chain≥C5 [5,10], but esters at C2 position are limited [11]. In our previous study, we have confirmed that the location of the hydroxy group is at C-2 of long-chain fatty acid by MS^n^ analysis [12]. Further successive loss of neutral molecules of CH2=CO (*m*/*z* 42 Da) and H_2_O (*m*/*z* 18 Da) from the fragment ion *m*/*z* 396 gives a molecular ion at *m*/*z* 354 and *m*/*z* 336, respectively. 

Another newly identified compound DMED-labeled-SFAHFA 3:0/20:0 (Figure 3b) shows the experimental mass at *m*/*z* 455.4198 (Theoretical *m*/*z*: 455.4213, mass error: –3.2 ppm) and lose the methyl radical to afford *m*/*z* 440. However, this peak is very weak in the MS spectrum. The most predominant ion is produced by the loss of dimethylamine moiety (*m*/*z* 45), which is identical to all other SFAHFAs. The loss of *m*/*z* 74 Da from the parent molecular ion suggests the acylated side chain of long-chain hydroxy fatty acid is propanoic acid. Furthermore, the loss of neutral molecules CH_2_CO and H_2_O from the fragment ion *m*/*z* 410 produces fragment ions at *m*/*z* 354 and *m*/*z* 336, respectively. The SFAHFAs (c), (h), (k) of Figure 3 and Figure 4 are the isomeric mixtures of acetic acid, and propanoic acid-containing SFAHFAs suggested by the loss of *m*/*z* 60 Da and *m*/*z* 74 Da in the same MS spectra, respectively. The limitation of our method is that even though DMED derivatization was employed and the acidic mobile phase was used, separation of isomers was not achieved. There could be a possibility that more appropriate columns and conditions or isotope labeling experiments [10] are necessary in order to enhance the separation of SFAHFAs structural isomers. Furthermore, the compounds, such as (l), (m), (n), (o), and (p) of Figure 4, were identified as SAHFA 24:1(Retention time (RT): 15.2 min, experimental *m*/*z*; 467.4197, theoretical *m*/*z*: 467.4213, mass error: −3.4 ppm), SAHFA 28:1 (RT: 18.2 min, experimental *m*/*z*; 523.4830, theoretical *m*/*z*: 523.4839, mass error: −1.7 ppm), SFAHFA 28:0 (RT: 18.9 min, experimental *m*/*z*; 525.4989, theoretical *m*/*z*: 525.4995, mass error: −1.1 ppm), SFAHFA 29:1 (RT: 18.6 min, experimental *m*/*z*; 537.4990, theoretical *m*/*z*: 537.4995, mass error: −0.9 ppm), and SFAHFA 29:0 (RT: 19.7 min, experimental *m*/*z*; 539.5150, theoretical *m*/*z*: 539.5152, mass error: −0.4 ppm) based on their retention time similarities and high mass accuracy results. 

To clarify, SFAHFAs are not artificial products of extraction and analysis, the DMED derivatized oleic-acid-d9 extracted ion chromatogram, and its mass spectra are shown in supporting information Figure S1. Further EICs of SFAHFAs in blank and oleic-acid-d9 esterified hydroxy fatty acids derived FAHFAs in samples were provided in Figure S2. The results, shown in Figure S2, clearly demonstrates that the SFAHFAs are not produced during extraction, and oleic-acid-d9 esterified hydroxy fatty acids derived FAHFAs are not detected in our samples. These results suggests that SFHAFAs are originally derived from the samples, not the artifacts. 

### 2.3. Fragmentation Pattern of DMED-labeled SFAHFAs

**Figure 5:**
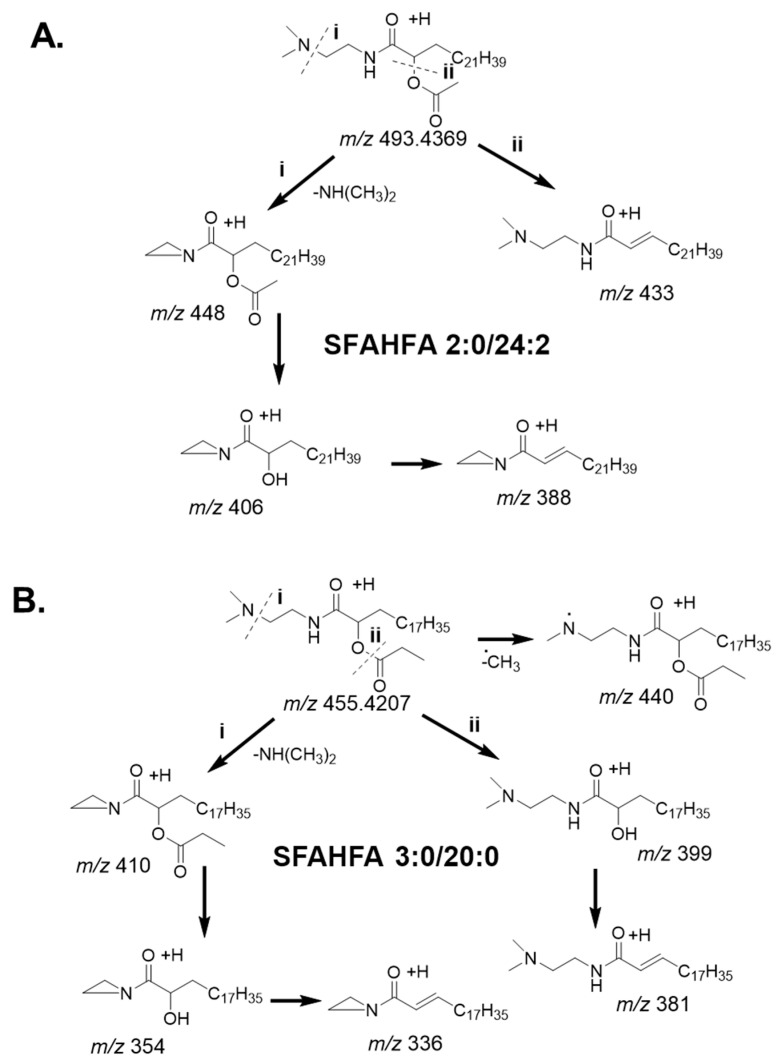
Plausible fragmentation pathway of DMED-labeled SFAHFA 2:0/24:2 (**A**) and SFAHFA 3:0/20:0 (**B**).

The plausible fragmentation patterns of newly identified and a representative DMED-derivatized SFAHFAs is shown in Figure 5. Figure 5A shows the fragmentation patterns of acetic acid esterified FAHFAs. The DMED-labeled-SFAHFA 2:0/24:0 gives molecular ion [M+H]^+^ at *m*/*z* 493.4352 in MS spectra. The fragment ion peak at *m*/*z* 448 was generated from the neutral loss of the dimethylamino group or –NH(CH_3_)_2_ (*m*/*z* 45 Da) of the molecular ion. The loss of dimethylamino moiety is commonly observed for the DMED-labeled FAHFAs [16], and it was observed to be the most predominant ion for all the identified SFAHFAs. The product ion at *m*/*z* 406 was annotated as the loss of a neutral ketene or CH_2_CO (*m*/*z* 42 Da) from the fragment ion *m*/*z* 448 [M+H–NH(CH_3_)_2_]^+^, which is normally observed for acetic acid derivatives [18]. Furthermore, the product ion at *m*/*z* 406 [M+H–NH(CH_3_)_2_–CH_2_CO]^+^ loses a neutral molecule of water (*m*/*z* 18 Da) to afford a fragment ion at *m*/*z* 338 [M+H–NH(CH_3_)_2_-CH_2_CO-H_2_O]^+^. Loss of water molecule is predominantly observed for saturated, less unsaturated and hydroxy fatty acids [19]. Alternatively, the loss of CH_3_COOH (*m*/*z* 60 Da) from the molecular ion confirms the acyl side chain of hydroxy fatty acid is acetate. Moreover, acetic acid esterified compounds generally produce a fragment ion by the loss of a neutral molecule of acetic acid by CID, which is evident in the literature [12,18].

The possible fragment ions of the newly identified compound SFAHFA 3:0/20:0 was shown in Figure 5B. The SFAHFA 3:0/20:0 as a molecular ion at *m*/*z* 455.4198 [M+H]^+^ in the MS spectra (Figure 3b). The fragment ion peak at *m*/*z* 440 is generated by the loss of the methyl radical of the molecular ion, which is more common in the CID spectra of N-methylated derivatives [20]. The product ion at *m*/*z* 410 was annotated as the loss of dimethylamino group or –NH(CH_3_)_2_ (*m*/*z* 45 Da) of the molecular ion. Consequently, *m*/*z* 354 represented the loss of neutral molecule ketene (CH_2_CO) from the most predominant fragment ion at *m*/*z* 410. Which further loses a neutral molecule of water to afford a peak at *m*/*z* 336. On the other hand, the molecular ion loses a neutral molecule of propanoic acid (CH_3_CH_2_COOH) to give a fragment ion at *m*/*z* 399, which indicates the presence of propanoic acid esterification at the hydroxy position of the SFAHFA. Subsequently, it loses a molecule of water to give fragment ion at *m*/*z* 381. A detailed investigation of the DMED-SFAHFAs structure revealed the reproducible fragmentation pattern for all compounds. In general, the loss *m*/*z* 60 is the characteristic fragment ion for acetic acid esters of hydroxy fatty acids, whereas the loss of *m*/*z* 74 for propanoic acid esters of hydroxy fatty acids.

### 2.4. Altered Levels of SFAHFAs as Possible Biomarkers of Obesity

**Figure 6:**
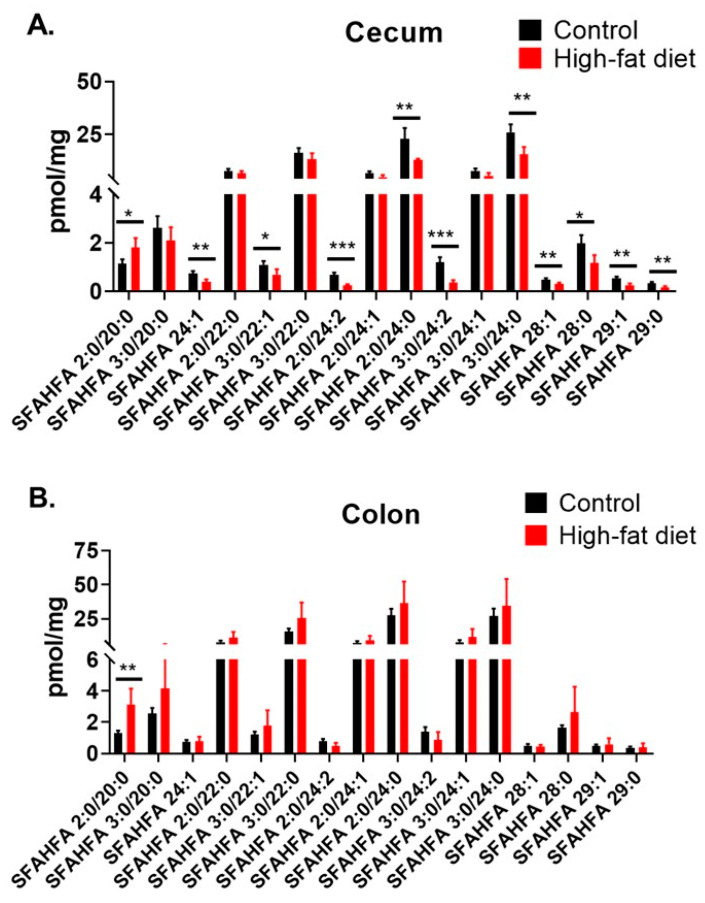
Amount of DMED-labeled SFAHFAs in contents of the cecum (**A**) and colon (**B**), of rats fed a normal diet (as controls) and HFD. (n = 6 each for cecum contents, n = 6 for control of colon contents and n = 3 for HFD group, *** *p* < 0.005, ** *p* < 0.05, * *p* < 0.1).

The relative concentrations of SFAHFAs in the cecum and colon contents of rats fed under HFD and compared with controls fed with normal diet (control) are shown in Figure 6. Our previous results suggested that an abundance of SFAHFAs majorly in colon and cecum contents [12]. Hence, in this study, we employed DMED-labeling of these samples to enhance their detection and to discover the new molecular species. Moreover, cecum and colon are abundant in gut microbiota and plausible sources of short-chain fatty acid or hydroxy fatty acid production [21]. The cecum content analysis results are shown in Figure 6A demonstrates the significantly decreased levels of several SFAHFAs under HFD condition. 

The comparison of total SFAHFA levels in the cecum and colon contents also shown a significant difference with a decreasing trend, especially in cecum contents (Figure S3). Many previous reports demonstrate the altered gut microbial composition under the HFD condition [22,23,24]. Besides, short-chain fatty acids are decreased in rats fed with HFD [25], and long-chain FAHFAs are reduced significantly in breastmilk of obese women [26] and serum of obese mice [5]. The decreased short-chain fatty acid levels and altered microbiota could plausibly account for the decreased SFAHFAs, however the detailed investigations are further necessary to confirm this hypothesis. Figure 6B shows the SFAHFAs levels in the colon contents, which are not significantly altered, suggesting that the cecum is a core region mainly affected by the HFD. Previous research suggests that HFD significantly reduces the cecal bacterial number [27]. Hence, there could be a possibility that bacteria or enzymes responsible for SFAHFA synthesis are reduced by the high-fat diet and hence, their derived SFAHFAs in the cecum. There is possible evidence that reported in the literature shows the participation of the microbiota in the metabolism of FAHFAs, for example, the examination of samples of meconium from healthy newborns [26], but detailed investigations are needed to understand these links and confirm the plausible biochemical pathways of SFAHFAs. 

## 3. Materials and Methods

### 3.1. Chemicals

Chromatographic grade methanol (MeOH), acetonitrile (CH_3_CN) isopropanol (IPA), chloroform (CHCl_3_), and 1M aqueous ammonium acetate solution (CH_3_COONH_4_) were purchased from Wako Pure Chemical Industries, Ltd., (Osaka, Japan). The deuterium-labeled internal standard Oleic acid-d9 is purchased from Avanti Polar Lipids (Alabaster, AL). All of the other reagents of reagent grade were obtained from Kanto Chemical Co., Inc. (Tokyo, Japan). N,N dimethylethylenediamine (DMED) was supplied by Sigma-Aldrich (MO, USA). 2-chloro-1-methylpyridinium iodide (CMPI) and triethylamine (Et_3_N) of analytical grade were obtained from Tokyo Chemical Industries Co., Ltd., (Tokyo, Japan). The reaction reagents, such as CMPI, Et_3_N, and DMED, were prepared in LC-MS grade CH_3_CN at 20 mM.

### 3.2. Animal Samples

The cecum and colon contents were collected (after exsanguination) from the two groups of 13-week-old WKAH/HkmSlc rats that were fed either a normal diet or high-fat diet fed. The details of animal experiments and diet compositions were provided in our previous report [12,28]. All the experiments were conducted by obtaining approval (No. 17-0050) from the Ethics Review Committee of Hokkaido University. 

### 3.3. Sample Preparation for LC-MS

Lipids from the cecum and colon contents were extracted by the Folch method [29] with minor modifications, established earlier in our laboratory [12]. The 17 µM of oleic acid (d9) was used as an internal standard during extraction. The concentrated lipid extracts were redissolved in 100 µL acetonitrile, and DMED labeling was carried out by the previously reported method with modifications [10]. The workflow of DMED labeling is shown in Figure 1A. In brief, to the total lipid extracts in CH_3_CN 20 µL of 20 mM Et_3_N, 10 µL of 20 mM CMPI, and 20 µL of 20 mM DMED were added and vortexed at 3500 rpm for 30 min at room temperature. Then, the derivatized extracts were concentrated in vacuum, redissolved in 100 µL methanol, and 10 µL of samples were injected into the LC-MS. The limitation of this method is that we avoided the long-time of derivatization or high temperatures do keep the SFAHFAs stable, as they are very susceptible to hydrolysis. Hence, there could be a possibility of poor efficiency. 

### 3.4. LC-MS Analysis 

The lipidomic analysis was performed using Shimadzu UHPLC coupled to LTQ-Orbitrap-MS by the method established earlier with minor modifications [12]. Chromatographic separation was achieved using Atlantic T3 C18 reverse-phase column (2.1 × 150 mm, 3 µm, Waters, Milford, MA, USA), maintained at 40 °C and a flow rate of 200 μL/min. The solvents used for elution consists of A: 10 mM aqueous CH_3_COONH_4_ having 0.1% acetic acid, B: IPA, and C: MeOH with a gradient flow of B(30%) and C(35%) at 1 min, B(75%) and C(15%) at 14 min, B(82.5%) and C(15%) at 27 min, B(82.5%) and C(15%) at 27 min and re-equilibrated for additional 3 min. The MS data were acquired in Electron spray ionization (ESI)-positive mode, with the capillary voltage (25 V), sheath gas flow (50 units), and the auxiliary gas (20 units), respectively. A Fourier Transform (FT) full scan range of *m*/*z* 100–800 was set to acquire MS^1^ spectra for high-resolution masses. The MS/MS spectra were obtained by data-dependent acquisition using collision-induced dissociation (CID) in the ion-trap mode for low-resolution masses at a collision energy of 40 V. The obtained raw data were processed by Xcalibur 2.2 (Thermo-Fisher Scientific Inc., San Jose, CA, USA) with a mass tolerance of 5.0 ppm and SFAHFAs are relatively quantified by using Oleic acid-d9. 

### 3.5. Statistical Analysis 

The results were visualized in Microsoft Excel 2016 and GraphPad Prism 8.0.1, as the mean ± SEM with applied one-tailed Student’s t-test with a *p*-values, *** *p* < 0.005, ** *p* < 0.05, * *p* < 0.1 are considered to be statistically significant. 

## 4. Conclusions

The chemical labeling of SFAHFAs using DMED slightly enhanced the detection of SFAHFAs, which led to the discovery of six new molecular species of acetic acid or propanoic acid esters of 2-hydroxy fatty acids (C ≥ 20) in the cecum and colon contents of WKAH/HKmSlc rats fed with normal and HFD. The MS/MS fragmentation pattern of DMED-labeled–SFAHFAs showed [M+H–45]^+^ as a characteristic ion in their spectra. SFAHFAs are significantly reduced in cecum contents of HFD group compared to controls. Further research is needed to establish absolute quantitative methods, understand sources, and biochemical actions of these novel lipids.

## Figures and Tables

**Table 1:** List of all the DMED-labeled-SFAHFAs identified with their exact masses.

SI No	Type of SFAHFA	Theoretical *m*/*z*	Experimental *m*/*z*	Mass Error ppm	Identified
1	SFAHFA 2:0/20:0	441.4056	441.4042	−3.1	[12]
2	SFAHFA 3:0/20:0	455.4213	455.4198	−3.2	This study
3	SFAHFA 24:1	467.4213	467.4197	−3.4	[12]
4	SFAHFA 2:0/22:0 or 3:0/21:0	469.4369	469.4355	−2.9	[12]
5	SFAHFA 3:0/22:1	481.4369	481.4355	−2.9	This study
6	SFAHFA 3:0/22:0	483.4526	483.4524	−0.4	[12]
7	SFAHFA 2:0/24:2	493.4369	493.4352	−3.4	This study
8	SFAHFA 2:0/24:1 or 3:0/23:1	495.4526	495.4507	−3.8	[12]
9	SFAHFA 2:0/24:0 or 3:0/23:0	497.4682	497.4677	−1.0	[12]
10	SFAHFA 3:0/24:2	507.4526	507.4514	−2.3	[12]
11	SFAHFA 3:0/24:1	509.4682	509.4673	−1.7	[12]
12	SFAHFA 3:0/24:0 or 2:0/25:0	511.4839	511.4833	−1.1	[12]
13	SFAHFA 28:1	523.4839	523.4830	−1.7	This study
14	SFAHFA 28:0	525.4995	525.4989	−1.1	[12]
15	SFAHFA 29:1	537.4995	537.4990	−0.9	This study
16	SFAHFA 29:0	539.5152	539.5150	−0.4	This study

## Supplementary Materials

The following are available online at https://www.mdpi.com/2218-1989/10/10/398/s1, Figure S1: Extracted ion chromatogram and mass spectra of internal standard oleic acid-d9, Figure S2: Extracted ion chromatograms (A) SFAHFAs in blank (B) Anticipated oleic acid-d9 esterified hydroxy fatty acid FAHFAs in sample (C) Anticipated oleic acid-d9 esterified hydroxy stearic acid FAHFAs in sample, Figure S3: Total SFAHFAs comparison between colon and cecum contents.
