# Supplementary material for: Chemical Labeling Assisted Detection and Identification of Short Chain Fatty Acid Esters of Hydroxy Fatty Acid in Rat Colon and Cecum Contents
Source: Metabolites. 2020 Oct 8;10(10):398. doi: 10.3390/metabo10100398 (PMC7600112; doi:10.3390/metabo10100398)
Supplement: Supplementary file 1 [file metabolites-10-00398-s001.pdf]

Figure S1:

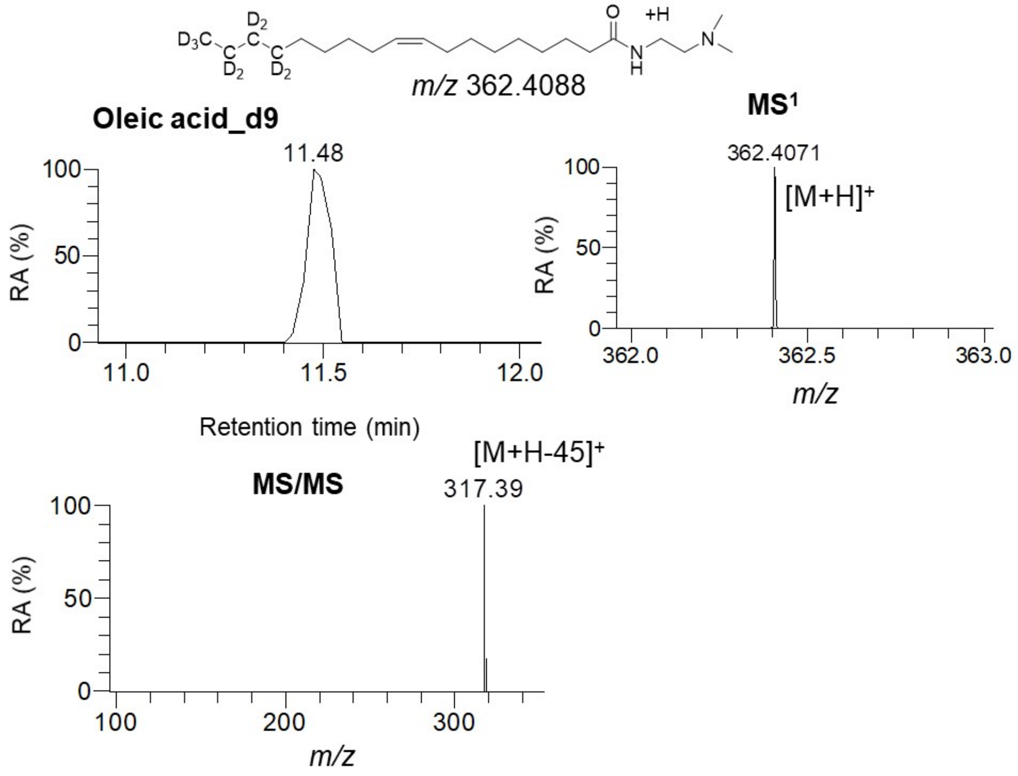

Figure S1: Extracted ion chromatogram and mass spectra of internal standard oleic acid-d9

Figure S2:

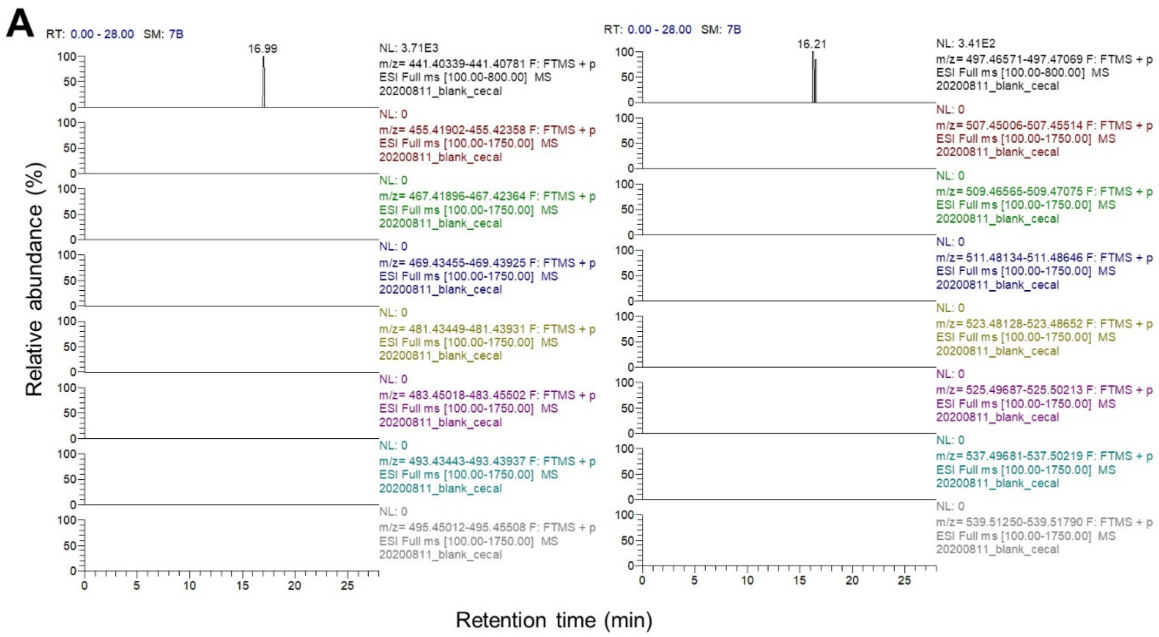

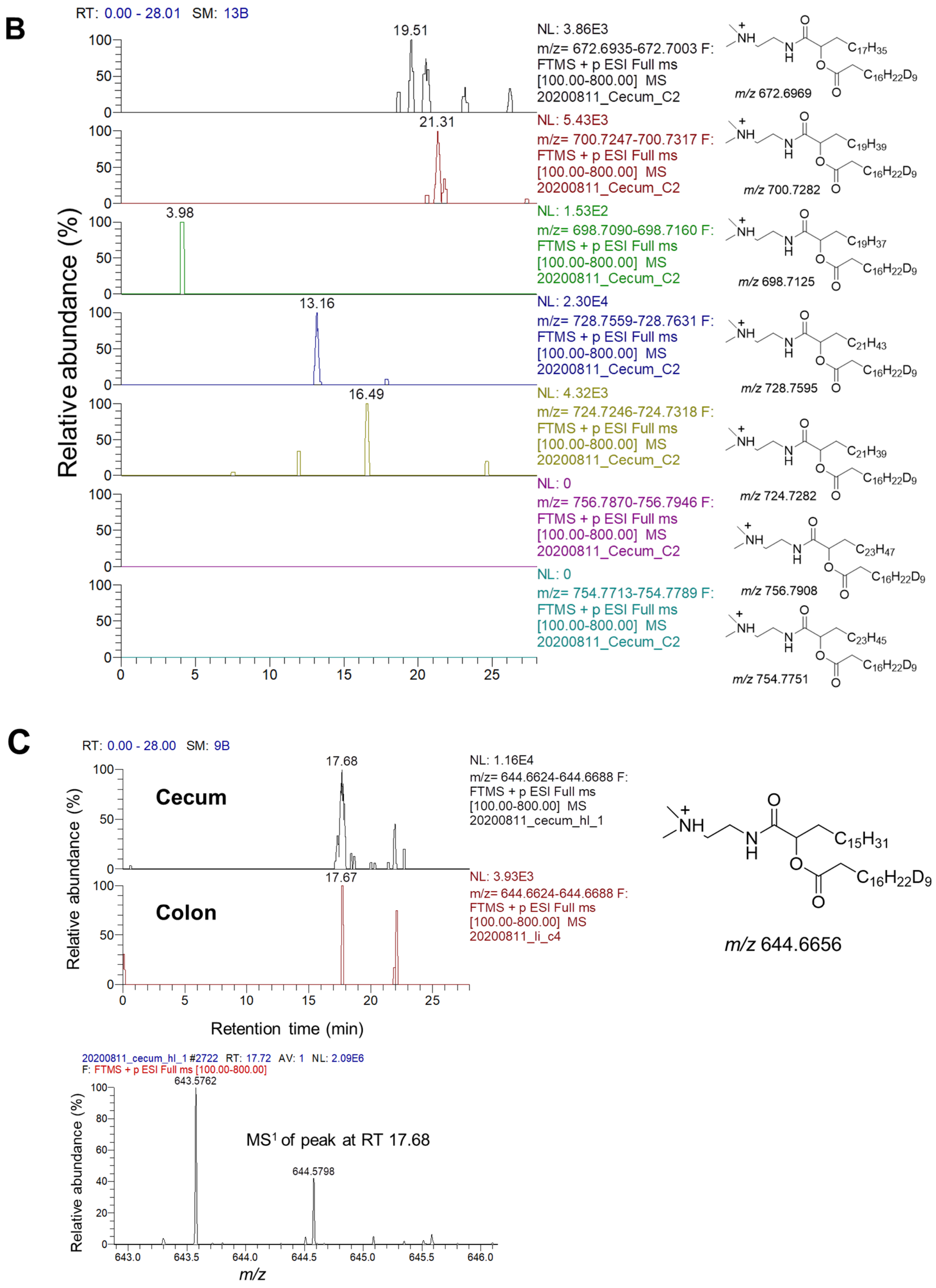

Figure S2: Extracted ion chromatograms **A.** SFAHFAs in blank **B**. Anticipated oleic acid-d9 esterified hydroxy fatty acid FAHFAs in sample **C.** Anticipated oleic acid-d9 esterified hydroxy stearic acid FAHFAs in sample.

Figure S3:

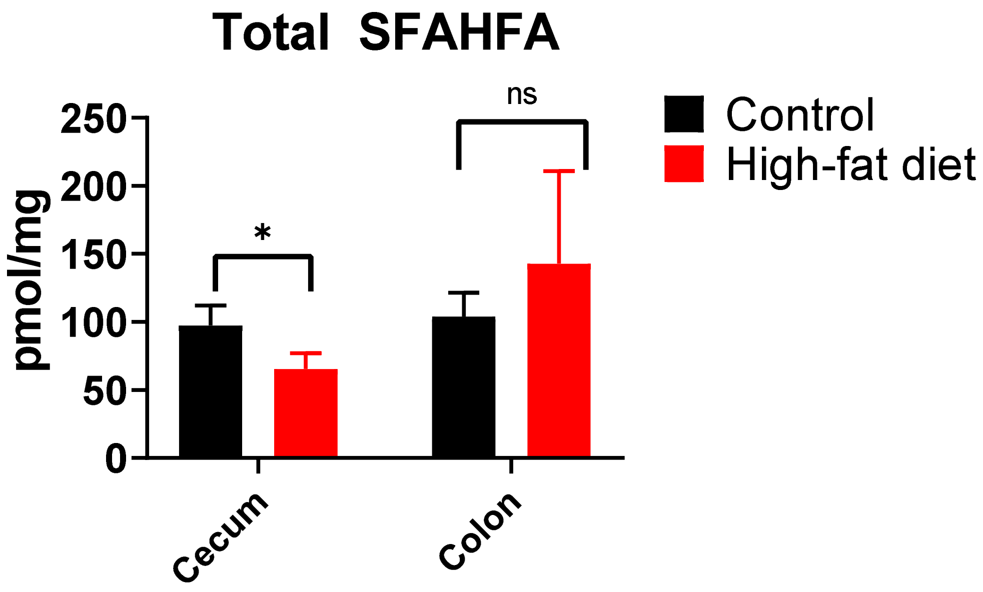

Figure S3: Total SFAHFAs comparison between colon and cecum contents.
